# Real-time effects of lateral positioning on regional ventilation and perfusion in an experimental model of acute respiratory distress syndrome

**DOI:** 10.3389/fphys.2023.1113568

**Published:** 2023-03-20

**Authors:** Mikuláš Mlček, João Batista Borges, Michal Otáhal, Glasiele Cristina Alcala, Dominik Hladík, Eduard Kuriščák, Leoš Tejkl, Marcelo Amato, Otomar Kittnar

**Affiliations:** ^1^ First Faculty of Medicine, Institute of Physiology, Charles University, Prague, Czechia; ^2^ Department of Anaesthesiology, Resuscitation and Intensive Medicine, First Faculty of Medicine, Charles University and General University Hospital in Prague, Prague, Czechia; ^3^ Pulmonology Division, Cardiopulmonary Department, Heart Institute, University of Sao Paulo, São Paulo, Brazil

**Keywords:** acute respiratory disease syndrome, mechanical ventilation, body position changes, ventilator-induced lung injury, lung collapse

## Abstract

Low-volume lung injury encompasses local concentration of stresses in the vicinity of collapsed regions in heterogeneously ventilated lungs. We aimed to study the effects on ventilation and perfusion distributions of a sequential lateral positioning (30°) strategy using electrical impedance tomography imaging in a porcine experimental model of early acute respiratory distress syndrome (ARDS). We hypothesized that such strategy, including a real-time individualization of positive end-expiratory pressure (PEEP) whenever in lateral positioning, would provide attenuation of collapse in the dependent lung regions. A two-hit injury acute respiratory distress syndrome experimental model was established by lung lavages followed by injurious mechanical ventilation. Then, all animals were studied in five body positions in a sequential order, 15 min each: Supine 1; Lateral Left; Supine 2; Lateral Right; Supine 3. The following functional images were analyzed by electrical impedance tomography: ventilation distributions and regional lung volumes, and perfusion distributions. The induction of the acute respiratory distress syndrome model resulted in a marked fall in oxygenation along with low regional ventilation and compliance of the dorsal half of the lung (gravitational-dependent in supine position). Both the regional ventilation and compliance of the dorsal half of the lung greatly increased along of the sequential lateral positioning strategy, and maximally at its end. In addition, a corresponding improvement of oxygenation occurred. In conclusion, our sequential lateral positioning strategy, with sufficient positive end-expiratory pressure to prevent collapse of the dependent lung units during lateral positioning, provided a relevant diminution of collapse in the dorsal lung in a porcine experimental model of early acute respiratory distress syndrome.

## Introduction

The acute respiratory distress syndrome (ARDS) is a frequent and important cause of morbidity and mortality in critically ill patients (Rubenfeld et al., 2005; Phua et al., 2009; Bellani et al., 2016). Mechanical ventilation in itself can harm the lung and cause the well-known ventilator-induced lung injury (VILI) (Slutsky and Ranieri, 2013; Borges et al., 2014), which can induce or aggravate ARDS. Some of the major mechanisms of VILI are known together as low-volume injury. Low-volume injury encompasses local concentration of stresses within and in the vicinity of collapsed regions in heterogeneously ventilated lungs, and also injurious cyclic recruitment of distal airways and alveoli. During mechanical ventilation, stress and strain are locally multiplied in a heterogeneously ventilated lung and VILI occurs at inhomogeneous interfaces (Cressoni et al., 2015; Cressoni et al., 2016). These mechanisms tend to predominate in more gravitational-dependent regions of the lungs and to occur in previously damaged lungs prone to collapse (Muscedere et al., 1994; Dreyfuss and Saumon, 1998; Otto et al., 2008).

The vertical gradient of pleural surface pressure is gravity dependent (Bryan et al., 1966). In supine body position lung collapse predominates within the lower (dorsal) half of the lungs, within the most gravitational-dependent units (Borges et al., 2006), where the transpulmonary pressure (*P*_L_ = airways pressure − pleural pressure) is the lowest. When such upper-lower (ventral-dorsal) lung zones inhomogeneous ventilation occurs in the supine position, as in most patients during early ARDS, a sequential lateral positioning strategy is conceivable. During the first side lateral positioning, the dorsal (dependent in supine) region from one of the lungs would be positioned more gravity-non-dependent and, consequently, its local *P*_L_ becomes selectively larger, potentially rendering recruitment of local collapsed lung units. During the other side lateral positioning, the same occurs with the contralateral lung. By doing that sequentially, such strategy may provide, after completed, a relevant diminution of dependent lung collapse.

A key component for the effectiveness of this sequential lateral positioning strategy is a proper real-time individualization of positive end-expiratory pressure (PEEP) whenever the lateral positioning is being applied. A sufficient and precisely titrated PEEP should be timely provided during each lateral posture to prevent collapse of the dependent lung units. Such PEEP individualization should be performed at the very beginning of each lateral period. The vertical gradient of *P*_L_, gravity dependent, changes with posture (D'Angelo et al., 1970), becoming greater when body position is changed from supine to lateral one (Agostoni et al., 1970). Compared to supine, the lateral posture by itself give rise to lower *P*_L_ in the most dependent units, since the distance between the upper most non-dependent units and the lower most dependent ones is longer in lateral than supine position. Without an appropriate PEEP titration, lateral positioning alone engenders lower *P*_L_ within the most gravitational-dependent lung units and increases the likelihood of their collapse.

Other potential effects of a lateral positioning strategy are changes on pulmonary blood flow distribution. For instance, a diminution of regional collapse may result in regional changes on local hypoxic pulmonary vasoconstriction efficiency; a modulation of regional overdistension may result in more or less diversion of pulmonary blood flow away from these units.

Electrical impedance tomography (EIT) is a non-invasive, radiation-free, real-time lung function imaging method. Cyclic variations in pulmonary air and blood content are the major determinants for the changes in thoracic impedance. Besides features like being a bedside imaging tool and providing the possibility of around-the-clock monitoring, the high temporal resolution is a crucial aspect of EIT imaging that allows for the study not only of ventilation distribution (Victorino et al., 2004), global and regional changes in lung volumes (Bachmann et al., 2018), but also of faster physiological phenomena, such as pulmonary perfusion distribution (Borges et al., 2012; Reinius et al., 2015; Reinius et al., 2019; Borges et al., 2020a; Borges et al., 2020b).

We thus aimed to study the real-time effects on ventilation and perfusion distributions, and on regional lung volumes, of a sequential lateral positioning strategy using EIT imaging in a porcine experimental model of early ARDS. Our hypothesis was that such strategy, including an appropriate and real-time personalization of PEEP whenever in lateral positioning, would provide attenuation of collapse in the dependent lung regions.

## Materials and methods

The study protocol was approved by the Institutional Animal Care and Use Committee of the First Faculty of Medicine, Charles University. The study was performed in an accredited animal laboratory of the Institute of Physiology, First Faculty of Medicine, Charles University, in accordance with Act No. 246/1992 Coll., on the protection of animals against the cruelty that is harmonized with EU legislation.

### Investigational protocol

All animals, lying in the supine position, received IV anesthesia using a combination of propofol, midazolam and fentanyl, and muscle relaxation using pancuronium, and were continuously monitored (physiological monitoring and measurements) as previously described (Popkova et al., 2020).

In all animals, baseline ventilation was delivered in volume-controlled ventilation, tidal volume (V_T_) ≤ 6 mL/kg, adjusted to a plateau pressure of ≤28 cmH_2_O and a driving pressure (ΔP) ≤ 15 cmH_2_O, fraction of inspired oxygen (F_I_O_2_) 1.0, inspiratory–expiratory ratio 1:2, respiratory rate 20–30 breaths/min (adjusted to end-tidal carbon dioxide <55 mmHg), and PEEP 10 cmH_2_O.

### ARDS experimental model

After physiological baseline measurements, we performed a two-hit injury model as previously described (Borges et al., 2014; Borges et al., 2015): repeated lung lavages with 30 mL/kg of warmed isotonic saline were applied until a partial pressure of arterial oxygen ratio (PaO_2_/F_I_O_2_) less than 150 mmHg was reached followed by 150 min of injurious mechanical ventilation using low PEEP and high inspiratory pressures (ΔP of 35 cmH_2_O). At the end of this period, we recorded a new set of physiological data.

After establishment of our experimental ARDS model, ventilation was delivered in volume-controlled ventilation, V_T_ 4–6 mL/kg, adjusted to a ΔP ≤ 15 cmH_2_O, F_I_O_2_ 1.0, inspiratory–expiratory ratio of 1:2, respiratory rate 30 breaths/min, and initial PEEP of 10 cmH_2_O.

### Sequential lateral positioning protocol

**FIGURE 1:**
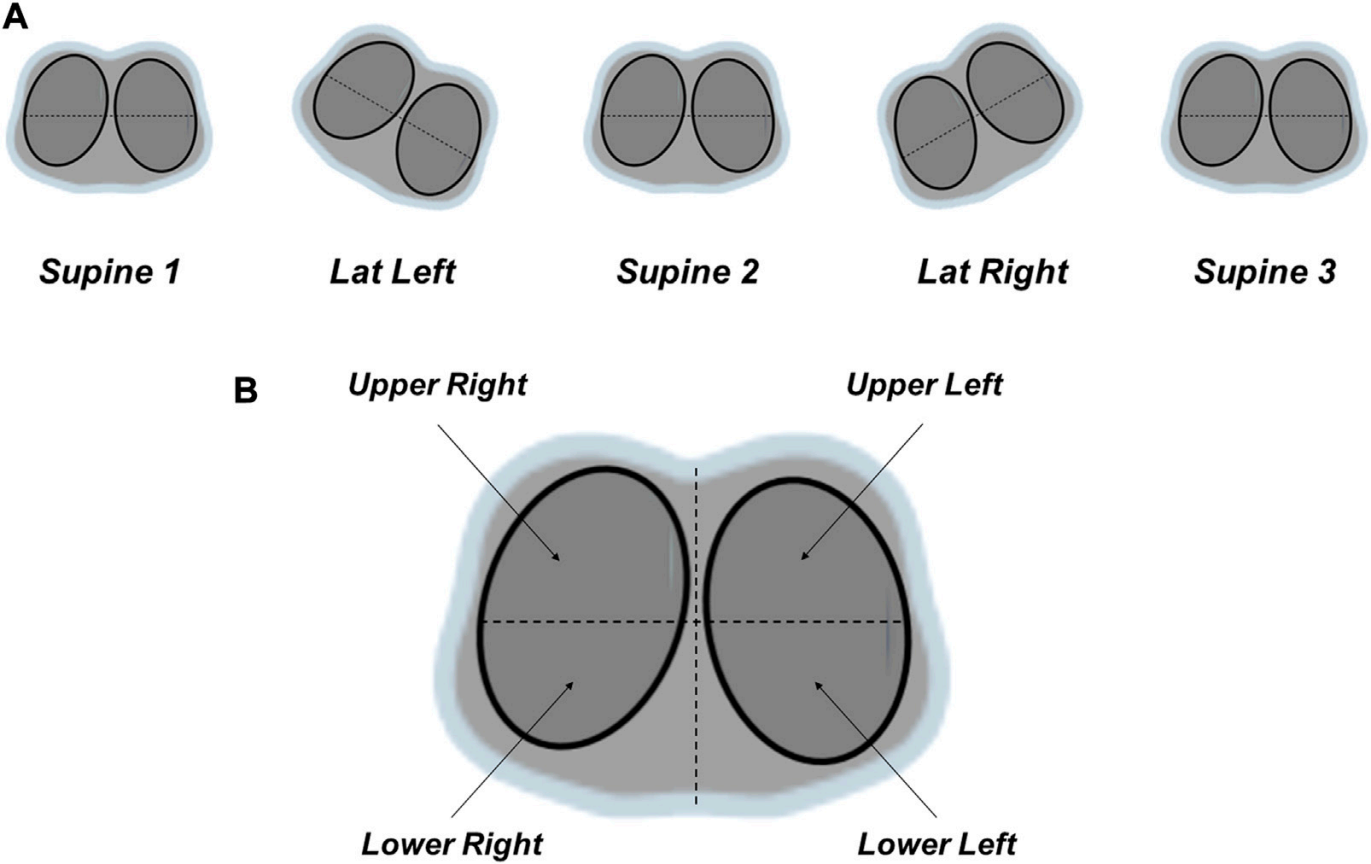
**(A)** Sequential lateral positioning strategy. All animals were studied in five body positions in a sequential order, 15 min each: Supine 1; Lateral Left (left lung positioned up); Supine 2 (after first lateral position); Lateral Right (right lung positioned up); Supine 3 (after second lateral position). Lateral positioning (30°) was performed with a platform-based rotation bed. **(B)** Regions-of-interest of the quantitative analysis of the functional images generated by electrical impedance tomography. The lungs were sub-segmented as the following: ventral (upper lung or anterior half = upper right + upper left quadrants), and dorsal (lower lung or posterior half = lower right + lower left quadrants); four quadrants: upper right, upper left, lower right, lower left.

All animals were studied in five body positions in a sequential order, 15 min each (Figure 1A): Supine 1; Lateral Left (left lung positioned up); Supine 2 (after first lateral position); Lateral Right (right lung positioned up); Supine 3 (after second lateral position).

Lateral positioning (30°) was performed with the platform-based rotation bed Multicare^®^ (LINET). At the end of each step (10 min), physiological and EIT measurements were acquired.

### EIT monitoring and measurements

Pulmonary EIT data were recorded at 50 Hz with 32 electrodes equidistantly placed around the circumference of the thorax just below the level of the axilla (Enlight, TIMPEL SA, São Paulo, Brazil) (Borges et al., 2012; Borges et al., 2020b).

The following functional images were generated by EIT:

1. Ventilation distributions and regional lung volumes: They were derived from relative impedance changes, which reliably track local, pixel-by-pixel changes in the content of air within the lung (Frerichs et al., 1998; Victorino et al., 2004). The ventilation distributions were expressed as the percentage of total pulmonary ventilation through each of the regions-of-interest (ROIs), total 100%.
2. Perfusion distributions: They were obtained by injecting a bolus of 10 ml of a hypertonic solution (NaCl 10%) into a central venous catheter during an expiratory breath hold for 20 s. Due to its high conductivity, NaCl 10% acts as an EIT contrast agent (Frerichs et al., 2002), which after injection into the right atrium during apnea passes through the pulmonary circulation, thereby producing a dilution curve that follows typical first-pass kinetics. The resulting regional impedance curves are then analyzed to quantitatively assess regional perfusion (Meier and Zierler, 1954; Thompson et al., 1964; Borges et al., 2012), expressed as the percentage of total pulmonary blood flow through each of the ROIs (total 100%).

For the ventilation distributions and regional lung volumes, the following EIT-derived parameters were continuously monitored and measured:

- Delta Z (∆Z): variation of impedance during a tidal breath, both global (surrogate of V_T_) and regionally (surrogate of regional V_T_ distribution).
- Delta end-expiratory lung impedance (∆EELI): variation of impedance plethysmography at end-expiration used as a surrogate of change in end-expiratory lung volume, both global and regionally.
- Distribution of regional tidal ventilation was determined as the relation of regional ΔZ/total ΔZ, expressed in percentage, and was also used to estimated regional V_T_ (V_Tr_) = (regional ΔZ/total ΔZ) x total V_T_.
- Regional lung compliance (C_Z_) was calculated as V_Tr_/ΔP.

For the quantitative analysis the lungs were sub-segmented into the following ROIs (Figure 1B):

- Ventral (upper lung or anterior half = upper right + upper left quadrants) and Dorsal (lower lung or posterior half = lower right + lower left quadrants).
- Four quadrants: upper right, upper left, lower right, lower left.

### Real-time individualization of PEEP during lateral positioning

The initial PEEP level was arbitrary set at 10 cmH_2_O. During each lateral positioning, at its beginning, PEEP level was titrated upwards, in incremental steps of 2 cmH_2_O, till all the following criteria were fulfilled:

- Global EELI stops decreasing.
- EELI of the dependent lung (left lung during Lateral Left position, right lung during Lateral Right position) stops decreasing after 2 min.
- EELI of the dependent lung (left lung during Lateral Left position, right lung during Lateral Right position) does not decrease by more than 0.5 to 1.0 times the ∆Z prior to lateralization of this dependent lung.

### Statistical analysis

The Shapiro-Wilk test was used to test data for normality. The one-way repeated measures analysis of variance (ANOVA) was used to determine whether there were any statistically significant differences between the population means of three or more levels of a within-subjects factor. The Bonferroni adjustment for multiple tests was applied for *post-hoc* comparisons. The paired-samples *t*-test was used to determine whether the mean difference between paired observations is statistically significantly different from zero. The statistical analyses were conducted with SPSS (version 25; IBM Corp, IBM SPSS Statistics for Windows, Armonk, NY). Individual *p*-values to indicate statistical tests’ significance are reported where relevant. Values presented are mean and SEM unless otherwise stated.

## Results

Seven crossbred (Landrace × Large White) healthy female pigs (*Sus scrofa domestica*), 6–7 months old, and weighting 69 ± 1.7 kg (mean ± SD) completed the whole study protocol and were included in these analyzes. All their data on ventilation distributions and regional lung volumes were available for analysis. In four animals, out of these seven, perfusion distributions during the sequential lateral positioning protocol were also acquired and were all available for analysis. We were able to complete the sequential lateral positioning protocol in all these seven pigs.

Hemodynamics was closely, online and continuously monitored throughout the study. In any moment during the sequential lateral positioning protocol, in none of the animals, hemodynamic instability occurred and it was a reason to interrupt the protocol.

**FIGURE 2:**
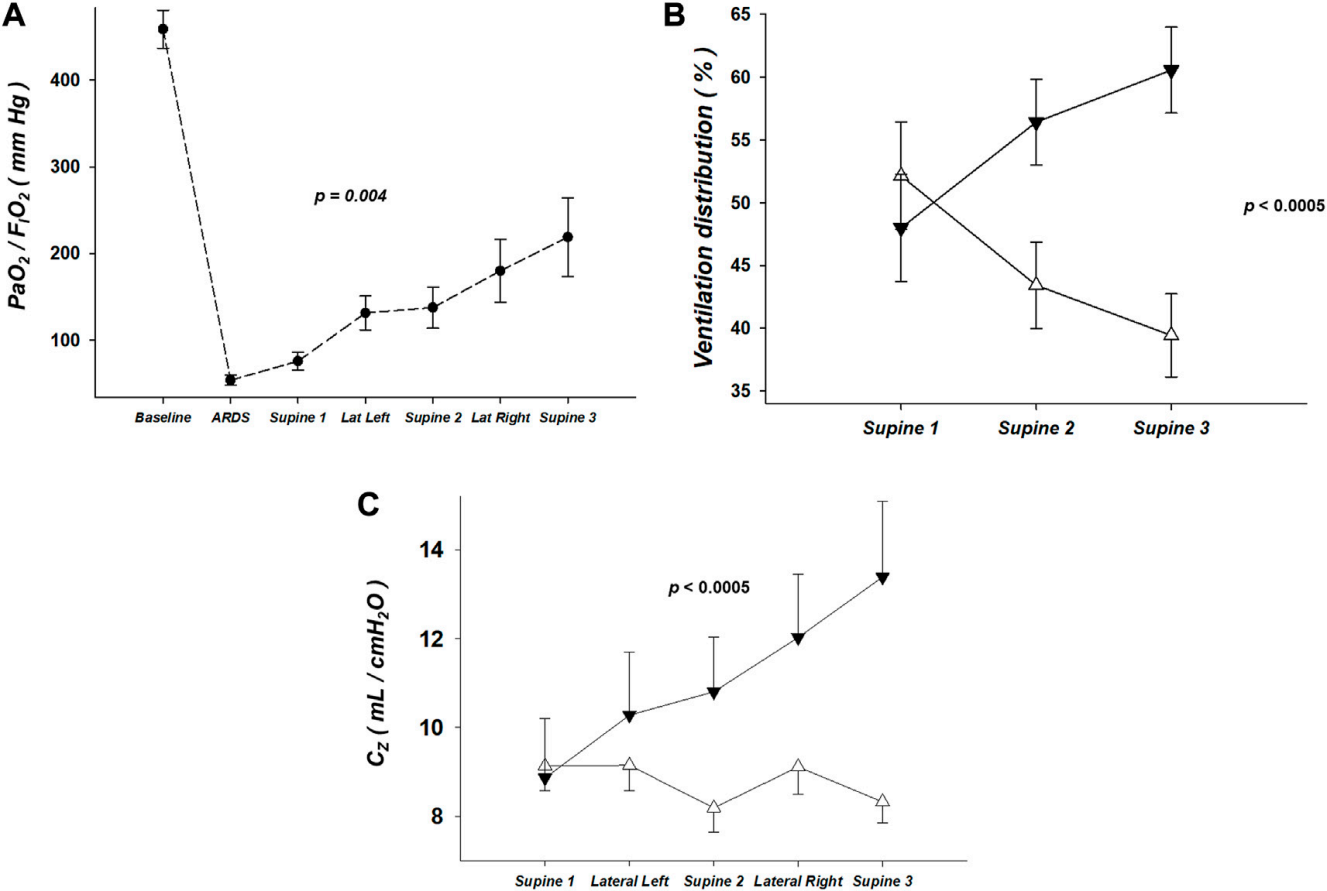
**(A)** Partial pressure of arterial oxygen ratio (PaO_2_/F_I_O_2_), expressed in units of mmHg, during all steps of the study. The induction of the two-hit experimental acute respiratory distress syndrome model resulted in a marked and expected fall in the oxygenation, and the sequential lateral positioning strategy resulted in a significant improvement of it. **(B)** Ventilation distribution, by electrical impedance tomography, of the regions-of-interest corresponding to the dorsal and ventral halves, expressed in units of percentage of total pulmonary ventilation (100%). Note that the ventilation distribution markedly changed along the sequential lateral positioning strategy: the % of ventilation of the dorsal half greatly increased when comparing the steps Supine 1, 2 and 3. Triangle up (white): Ventral half. Triangle down (black): Dorsal half. **(C)** Regional lung compliance (C_Z_), by electrical impedance tomography, of the regions-of-interest corresponding to the dorsal and ventral halves, expressed in units of mL/cmH_2_O. Note that the C_Z_ of the dorsal half substantially increased along the sequential lateral positioning strategy. And that occurred without causing significant decrease of the C_Z_ of the upper half. Triangle up (white): Ventral half. Triangle down (black): Dorsal half.

Figure 2A shows the PaO_2_/F_I_O_2_ evolution during all steps of the study. The induction of our experimental ARDS model resulted in a marked and expected fall in the oxygenation, and the sequential lateral positioning strategy resulted in a significant improvement of it.

Table 1 shows the measured values (mean ± SD) of PEEP, respiratory system compliance, ΔP, and V_T_ at the end of all steps of the sequential lateral positioning strategy.

**TABLE 1:** Mechanical ventilation parameters and respiratory system mechanics.

	Supine 1	Lateral left	Supine 2	Lateral right	Supine 3
Positive end-expiratory pressure (cmH_2_O)	10.6 ± 1.2	12.9 ± 1.7	12.9 ± 1.6	14.7 ± 2.0	14.7 ± 2.0
Respiratory System Compliance (mL/cmH_2_O)	18.0 ± 3.5	19.4 ± 3.7	19.0 ± 3.3	21.1 ± 3.7	21.7 ± 4.5
Driving Pressure (cmH_2_O)	19.7 ± 5.5	18.1 ± 4.7	18.8 ± 5.0	16.3 ± 4.2	16.6 ± 4.8
Tidal Volume (mL)	351.0 ± 100.5	345.4 ± 84.5	352.4 ± 85.8	340.4 ± 82.3	349.4 ± 77.2

The ventilation distribution markedly changed along the sequential lateral positioning strategy (Figure 2B). The % of ventilation of the *Dorsal* half greatly increased when comparing the steps Supine 1, 2 and 3 (Figure 2B). Likewise, the C_Z_ of the *Dorsal* half substantially increased along the sequential lateral positioning strategy (Figure 2C).

**FIGURE 3:**
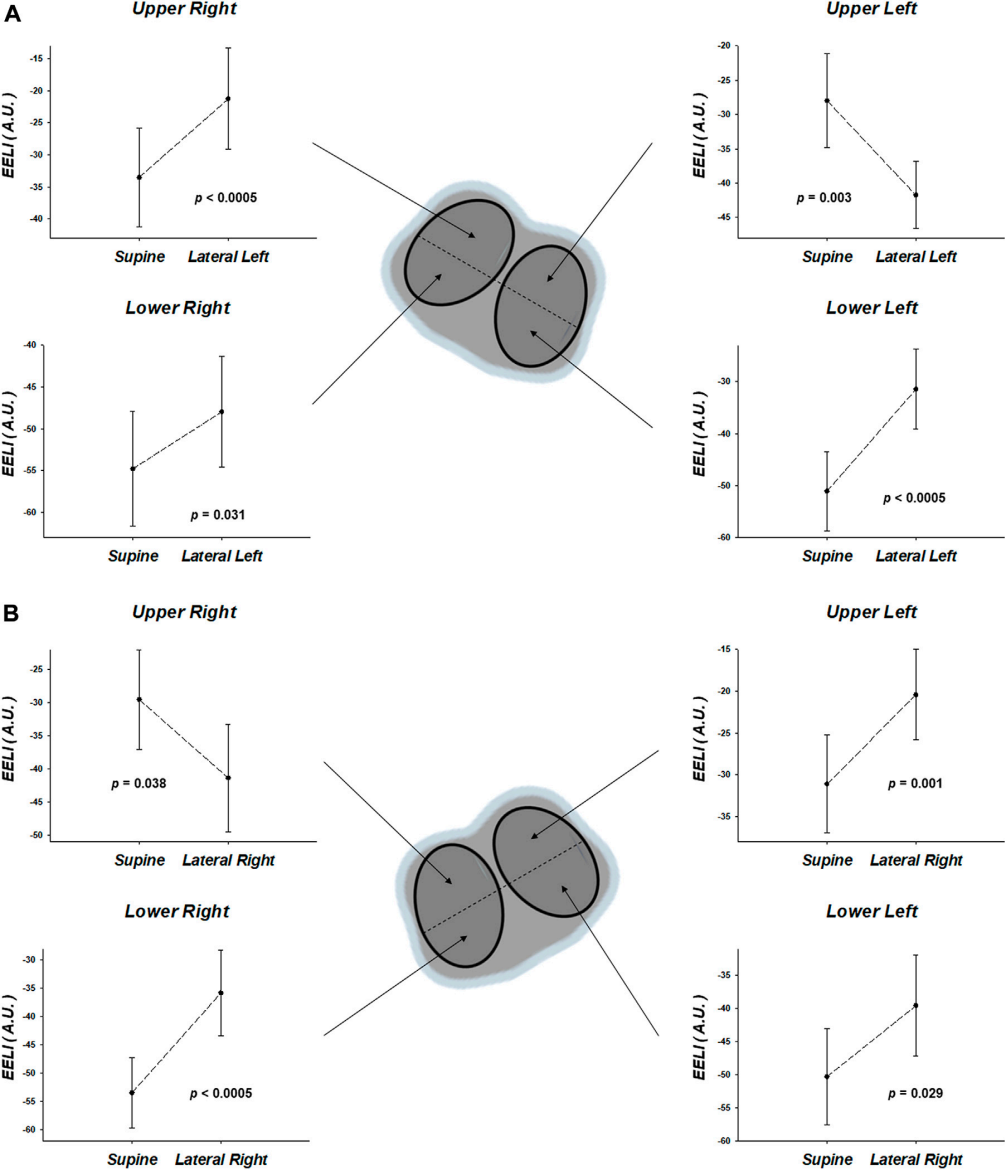
Regional end-expiratory lung impedance (EELI), by electrical impedance tomography, of the regions-of-interest corresponding to quadrants. The results, expressed in units of arbitrary units (A.U.), are the comparisons between each lateral position with the immediate previous supine position [**(A)**, supine vs. lateral left; **(B)**, supine vs. lateral right]. Note that the EELI in lateral position, regardless of the lateralized side, comparing with the immediate previous supine position, showed an increase of EELI in the two quadrants of the non-dependent lung (right lung during lateral left position, left lung during lateral right position). In contrast, the two quadrants of the dependent lung showed changes of EELI in opposite directions, with a decrease in the ventral and an increase in the dorsal quadrant.

The analysis of the lungs divided by quadrants in the lateral position, regardless of the lateralized side, comparing with the immediate previous supine position (Figures 3A, B), showed an increase of EELI in the two quadrants of the non-dependent lung (right lung during lateral left position, left lung during lateral right position). In contrast, the two quadrants of the dependent lung showed changes of EELI in opposite directions, with a decrease in the ventral and an increase in the dorsal quadrant.

**FIGURE 4:**
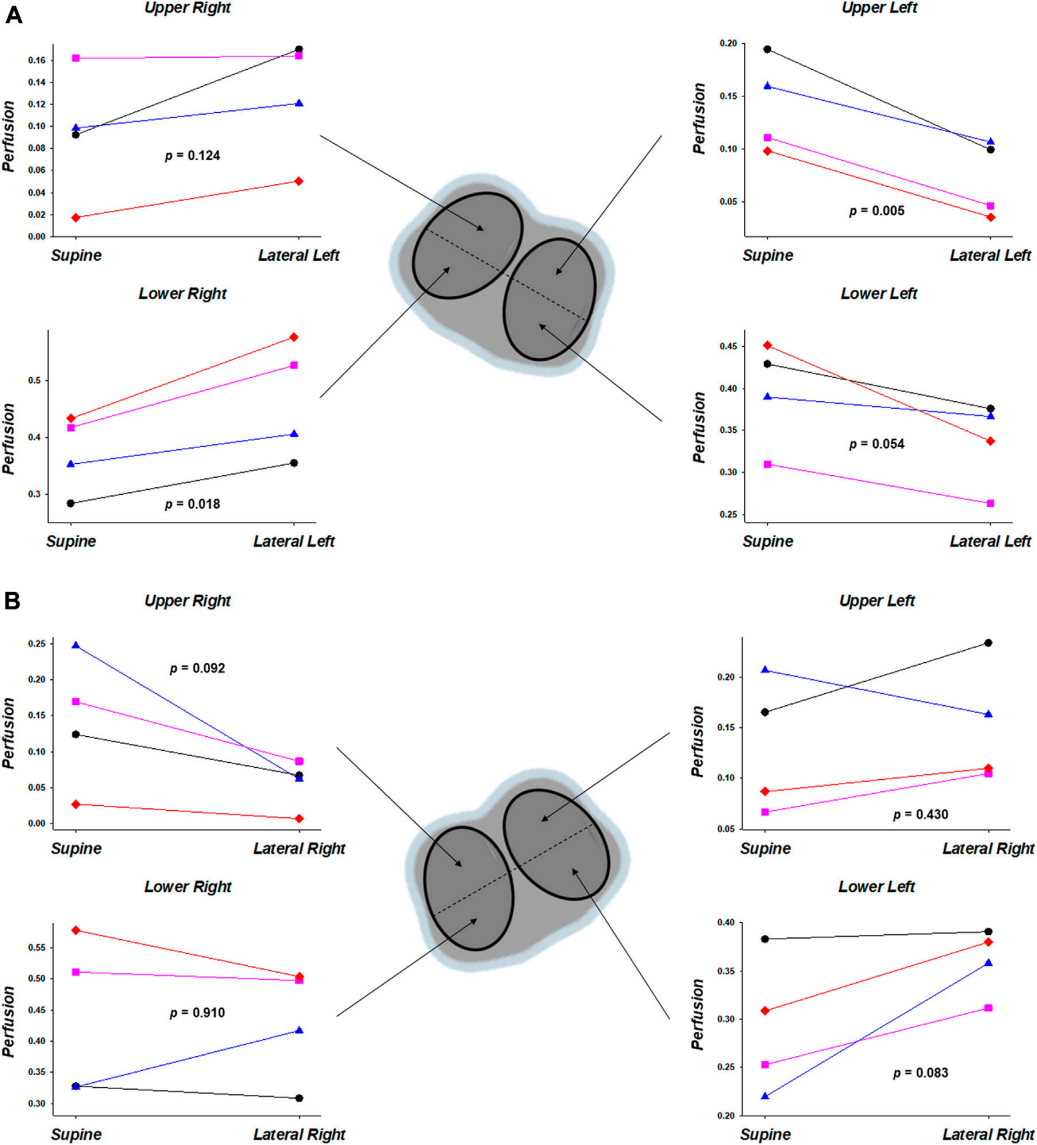
Regional pulmonary perfusion, by electrical impedance tomography, of the regions-of-interest corresponding to quadrants. The results (*n* = 4), expressed in units of decimals of total pulmonary perfusion (1.0), are the comparisons between each lateral position with the immediate previous supine position [**(A)**, supine vs. lateral left; **(B)**, supine vs. lateral right]. Note that the analysis of the lungs divided by quadrants in the lateral position, regardless of the lateralized side, comparing with the immediate previous supine position, showed an increase of perfusion in the two quadrants of the non-dependent lung and a decrease of perfusion in the two quadrants of the dependent lung. Some of these findings did not reach statistical significance.

Regarding pulmonary perfusion distribution, the analysis of the lungs divided by quadrants in the lateral position, regardless of the lateralized side, comparing with the immediate previous supine position (Figures 4A, B), showed an increase of perfusion in the two quadrants of the non-dependent lung and a decrease of perfusion in the two quadrants of the dependent lung. Some of these findings did not reach statistical significance (Figures 4A, B).

## Discussion

The induction of our two-hit experimental ARDS model (Borges et al., 2014; Borges et al., 2015; Russ et al., 2021) resulted in a marked fall in oxygenation along with low regional ventilation and compliance of the dorsal half of the lung (gravitational-dependent in supine position). Both the regional ventilation and compliance of the dorsal half of the lung greatly increased along of the sequential lateral positioning strategy, and maximally at its end. In addition, a corresponding improvement of oxygenation occurred. These findings, altogether, strongly point out that the sequential lateral strategy resulted in a significant recruitment of units located in the dorsal half of the lung. Importantly, that occurred without causing significant simultaneous overdistension of the upper half, as suggested by no relevant decrease of the regional compliance of the ventral half during the sequential lateral protocol (Figure 2C).

One may argue that these results may be more related to the PEEP changes and or to the effect of time than resulting from the sequential lateral strategy. But the increments on PEEP level were only approximately 2–4 cmH_2_O (Table 1), which resulted in inspiratory airway pressures (Table 1) far below the needed ones to reach the critical opening pressures of the most gravitational-dependent units located in the dorsal half of ARDS lungs in supine position (Borges et al., 2006; de Matos et al., 2012). In addition, these small-scale increments on PEEP were not associated with any recruitment maneuver using brief increases in airway pressures. Regarding a potential time effect, although it is well known that a one-hit injury model consisting of only repeated lung lavages is rapidly reversible (Huber et al., 1966; Kloot et al., 2000; Ballard-Croft et al., 2012; Russ et al., 2021), that is not the case for our two-hit injury model (Borges et al., 2014; Borges et al., 2015; Russ et al., 2021).

A randomized and controlled trial demonstrated the feasibility and efficacy of a postural recruitment maneuver in children with anesthesia-induced atelectasis (Acosta et al., 2020). Besides being applied in children with healthy lungs, another important difference between the Acosta et al. and our study is the lack of any real-time PEEP personalization. Zhao et al. (2021) reported the use of EIT for individualized mechanical ventilation and body positioning strategies in one patient with COVID-19-related ARDS. They provided a continuous lateral rotation therapy (Staudinger et al., 2010) to the patient. Lateral (both left and right, ∼40° with pillows) and supine positions were performed and resulted in a feeble increase in oxygenation. Similarly to the study in children with anesthesia-induced atelectasis (Acosta et al., 2020), a key difference between the Zhao et al. case report and our study is the lack of a strategy to personalize PEEP during the lateral positioning. Very recently, Roldan et al. (2022) reported an exploratory study in early mechanically ventilated patients with COVID-19-related ARDS, in which a sequential lateral positioning was applied as a lung recruitment maneuver. But they chose between only two PEEP levels using the one-breath decremental PEEP maneuver to calculate the recruitment-to-inflation ratio (R/I ratio) (Chen et al., 2020): PEEP = 15 cmH_2_O for R/I ratio > 0.5, and PEEP = 12 cmH_2_O for R/I ratio ≤ 0.5. In addition, choosing the PEEP level based on the R/I ratio has a key limitation: lung recruitability is tested over a fixed change in PEEP and the lack of a wider range of PEEP is a limitation to completely assess the potential for recruitment. Furthermore, Roldán et al. study lacks a personalized PEEP titration when in lateral position, unlike we did in the present study. The individualization of PEEP is essential to avoid dependent lung collapse during lateral positioning and to keep open the non-dependent lung when returning to the supine position.

The vertical gradient of *P*_L_, which is mainly due to gravity, changes with body mass and posture (D'Angelo et al., 1970). Agostoni et al. (1970) showed that the *P*_L_ gradient increases when body position is changed from supine to lateral position. They demonstrated that lateral position leads to higher *P*_L_ in the most non-dependent units and lower *P*_L_ in the most dependent ones. That is mainly because the thoracic right-to-left distance is longer than the anterior–posterior. Thus, generally, without an appropriate PEEP personalization, lateral positioning alone increases heterogeneity of *P*_L_ across the parenchyma. Notwithstanding that, the local and net effects of lateral positioning depend on PEEP. A key component of our sequential lateral positioning strategy was a proper real-time individualization of PEEP whenever the lateral positioning was applied, providing a sufficient PEEP to prevent collapse of the dependent lung units when in the lateral position while a simultaneous higher *P*_L_ in the most non-dependent units would be potentially able to recruit collapsed lung units. A key concept here is that this PEEP individualization should be performed at the very beginning of each lateral period. Only by doing that, this titrated PEEP can be able to prevent the collapse of the dependent units when in lateral position. We think that our findings as a whole suggest that our lateral positioning with real-time individualization of PEEP attained the two discussed purposes, promoting—during the lateralization—lung recruitment in the most non-dependent regions while preventing lung collapse in the most dependent ones.

The EELI analysis of the lungs divided by quadrants in the lateral position, regardless of the lateralized side, comparing with the immediate previous supine one, showed a predictable increase of EELI in the two quadrants of the non-dependent lung (Figures 3A, B). But, in contrast, the two quadrants of the dependent lung showed changes of EELI in opposite directions, with an anticipated decrease in its ventral but an unexpected increase in its dorsal quadrant (Figures 3A, B). Similar findings were reported by Roldan et al. (2022). The authors discuss (Roldan et al., 2022) that a combination of effects could explain this paradoxical finding in the most dependent lung quadrant in the lateral position, including an increase in the distance between the upper most non-dependent lung units and the lower most dependent ones, which is longer in lateral than supine position (D'Angelo et al., 1970), the overlying weight of the heart and mediastinum, and the limitation of thoracic expansion in the lateral position. We hypothesize that an additional factor underlying this paradoxical finding in the most dependent lung quadrant in the lateral position may be related to distinct local effects of abdominal contents on regional lung volumes. Since the vertical gradient of pleural surface pressure is gravity dependent (Bryan et al., 1966) and the abdomen is denser and more fluid-like than the lung, the abdomen in the horizontal postures should be important in originating this gradient. Agostoni et al. (1970) studied the effect of the abdomen on the vertical gradient of pleural surface pressure in different body positions by determining the topography of pleural surface pressure after evisceration. Their data showed that the vertical gradient of pleural surface pressure does not depend essentially upon the density of the lung and that part of it depends upon the abdomen. They found that in supine, prone and lateral (90°) positions the overall vertical gradient of *P*_L_ after evisceration decreased 2-3 times, and more than the lung density. Their results clearly indicated that the vertical gradient of *P*_L_ is markedly affected by the vertical gradient of abdominal pressure. The evisceration produced an increase of lung volume that was greatest in the supine posture, medium in the lateral posture and almost negligible in the prone posture. But they were not able to scrutinize regional effects. We plan to carry on this line of research and to perform a specific experimental study to test our hypothesis regarding local effects of abdominal contents on regional lung volumes in supine vs. lateral body position (30°), with and without evisceration.

Distinct abnormalities of the pulmonary blood flow distribution have been described in ARDS and there are conflicting data (Dantzker et al., 1979; Pistolesi et al., 1986; Schuster and Haller, 1990; Dakin et al., 2011). Studying the effects of nitroprusside and or prostacyclin on regional pulmonary perfusion after oleic acid-induced acute pulmonary edema (Schuster and Haller, 1990), Schuster et al. evidenced that blood vessels in edematous lung regions remained vasoreactive only until derecruited. The authors discuss that the derecruitment process involves an interaction between edema accumulation and vasoconstriction, in which the actual pattern of regional pulmonary perfusion after lung injury represents a balance between mechanisms responsible for vascular derecruitment and vasodilation from prostacyclin production (Schuster and Haller, 1990). Pistolesi et al. studied ARDS patients and found focal perfusion defects, mostly peripheral and dorsal, and perfusion redistribution both from base to apex and in the dorsoventral direction (Pistolesi et al., 1986). Their redistribution data better correlated with mean pulmonary arterial pressure and vascular resistance. Dakin et al. (2011) investigated changes in regional perfusion and tissue distribution in ARDS patients in comparison with healthy subjects. They concluded that perfusion of collapsed alveoli is greatly variable between individuals with ARDS, presenting a distribution that cannot be explained by gravity alone. Another study using an oleic acid lung injury model showed an only slight correlation between improvement of oxygenation and perfusion redistribution, suggesting that mechanisms other than hypoxic vasoconstriction may affect regional perfusion after lung injury (Schuster and Marklin, 1986). Importantly, the studies above had no data on supine vs. lateral body position. We analyzed the real-time effects on perfusion distributions of supine vs. lateral positioning. Interestingly, we found an increase of perfusion in the two quadrants of the non-dependent lung and a decrease of perfusion in the two quadrants of the dependent lung. That was found regardless of the lateralized side, comparing with the immediate previous supine. Besides the factors discussed above, which may had played major or partial roles, each factor more or less important depending on lung region and local lung condition, we must consider too a particular contribution of the normally aerated/ventilated regions of the lung. The observed changes in perfusion induced by posture and PEEP can also be partly explained by alterations occurring in these normally aerated portions of the pulmonary vasculature, following the West’s zone model (West et al., 1964). Another theoretical explanation for such non-gravitational effect could be a more efficient local hypoxic pulmonary vasoconstriction. But the changes we found in the ventilation and compliance maps point out for an ongoing recruitment, during lateral position, of previously collapsed lung units in supine position. Patently, further studies are needed to better understand these findings, also due to the small number of animals with perfusion data.

Regarding regional lung compliance by EIT (C_Z_), we would like to point out the following: by having pleural sensors located at the ventral, mid and dorsal positions of the thorax (Bastia et al., 2021; Katira et al., 2021), it was observed that the absolute pleural pressures are substantially different across different regions of the lung. The swings in pleural pressure, and consequently the delta-transpulmonary driving pressures, however, are surprisingly similar (Katira et al., 2021). The exception to this condition is during the occurrence of pendelluft (Yoshida et al., 2013), which is a dynamic condition during lung inflation. If we measure driving transpulmonary pressures during quasi-static conditions, after complete end-inspiration, they are quite similar.

There are several limitations in the present study. Firstly, we included a small number of animals and must thus be considered an exploratory and descriptive study. Changes were assessed after 10 min in each body position, but the optimal duration of each step is unknown, and thus, merits further study as well as testing the duration of the benefits observed. We used a sequential order rather than a randomized order. Another limitation is the lack of the following assessment within all the animals, each animal serving as its own control: a comparison between the sequential lateral position protocol (with its real-time individualizations of PEEP when in lateral positions, at their beginnings), with an exactly same sequence (same time and PEEP levels of the individualizations in lateral), keeping all the same mechanical ventilation settings, but without body position changes (whole sequence in supine). Finally, apart from PEEP, the other optimum ventilator settings during the sequential lateral positioning strategy are unknown and should be tested in future studies.

## Conclusion

A sequential lateral positioning strategy, with a real-time individualization of PEEP to prevent collapse of the dependent lung units whenever in the lateral position, provided a relevant diminution of collapse in the dorsal lung in a porcine experimental model of early ARDS. This physiology-based, personalized, simple and gentle strategy may afford a relevant diminution of lung collapse and, consequently, it holds a promising potential for decreasing major mechanisms of VILI.

## Data Availability

The raw data supporting the conclusion of this article will be made available by the authors, without undue reservation.
