# Corrigendum: Real-time effects of lateral positioning on regional ventilation and perfusion in an experimental model of acute respiratory distress syndrome

**DOI:** 10.3389/fphys.2023.1215839

**Published:** 2023-06-20

**Authors:** Mikuláš Mlček, João Batista Borges, Michal Otáhal, Glasiele Cristina Alcala, Dominik Hladík, Eduard Kuriščák, Leoš Tejkl, Marcelo Amato, Otomar Kittnar

**Affiliations:** ^1^ First Faculty of Medicine, Institute of Physiology, Charles University, Prague, Czechia; ^2^ Department of Anaesthesiology, Resuscitation and Intensive Medicine, First Faculty of Medicine, Charles University and General University Hospital in Prague, Prague, Czechia; ^3^ Pulmonology Division, Cardiopulmonary Department, Heart Institute, University of Sao Paulo, São Paulo, Brazil

**Keywords:** acute respiratory distress syndrome, mechanical ventilation, body position changes, ventilator-induced lung injury, lung collapse

In the published article, there was an error in the **Funding** statement. The **Funding** information was missing in the original article. The correct **Funding** statement appears below.

